# Genetic Variants in *SPINK1*, *PRSS1,* or *CFTR* Are Not Associated With The Development of Post-ERCP Pancreatitis

**DOI:** 10.1097/MPA.0000000000002465

**Published:** 2025-01-16

**Authors:** Mike J.P. de Jong, Roel C.M. van Aard, Romy N. Kuipers, René H.M. te Morsche, Foke van Delft, Peter D. Siersema

**Affiliations:** * Department of Gastroenterology and Hepatology, Radboud University Medical Center, Nijmegen; † Department of Research and Development, St. Antonius Hospital, Nieuwegein; ‡ Department of Gastroenterology and Hepatology, Erasmus MC—University Medical Center, Rotterdam, The Netherlands

Post-endoscopic retrograde cholangiopancreatography (ERCP) pancreatitis (PEP) occurs with an incidence of ∼10%.^1^ Although the majority of PEP cases are classified as mild to moderate, 0.5% of patients undergoing ERCP develop a severe disease course, and 0.2% die because of PEP.^1^ Despite administration of a diclofenac suppository before ERCP and insertion of a pancreatic duct stent following unintentional cannulations of the pancreatic duct as PEP prophylaxis, PEP still occurs following ERCP.^2^

Patients with a history of PEP have an increased risk of developing subsequent episodes of PEP with an odds ratio (OR) of up to 8.7.^2^ Individual genetic susceptibility for the development of acute pancreatitis might be attributed to this increased risk. Associations between variants in pancreatitis-associated genes and the susceptibility to acute pancreatitis have been documented. The most well-known genes and their variants are cystic fibrosis transmembrane regulator (*CFTR* p.F508del, p.R117H, p.R75Q), protease serine 1 (*PRSS1* p.R122H, p.N29I), and serine protease inhibitor Kazal-1 (*SPINK1* p.N34S, p.P55S).^3–7^

This study aims to investigate whether patients who developed PEP were more frequently carriers of the aforementioned genetic variants. We hypothesize that identifying patient-specific genetic variants could contribute to the development of tailored prophylactic strategies for PEP management.

This multicenter cohort study was designed as a substudy of the G-PEP study, which aimed to investigate potential differences in the presence of single nucleotide polymorphisms (SNPs) in cytochrome P450 (CYP) enzymes involved in the diclofenac metabolism (unpublished data, ClinicalTrials.gov NCT05267379). After ethical approval from the Radboudumc Medical Ethical Board, Nijmegen, the Netherlands (NL68494.091.18), this study was carried out in 3 community and 1 tertiary academic hospital in the Netherlands. Patients aged 18 years or older who underwent ERCP between 2012 and 2022 were screened for eligibility. Exclusion criteria included the diagnosis of pancreatic cancer, acute or chronic pancreatitis, contraindications for administration of nonsteroidal anti-inflammatory drugs (NSAIDs), or the presence of a biliodigestive anastomosis. Eligible patients received an information letter, informed consent form, and buccal smear kit at home. After obtaining a signed informed consent form and a buccal smear, patients were divided into either the PEP or non-PEP group. PEP was assessed according to the Cotton criteria. The aim was to include over 100 PEP patients and at least twice as many patients without PEP. Baseline and ERCP characteristics were collected from electronic health records, and data was double-checked by a second researcher. The study adhered to the Declaration of Helsinki and complied with the Dutch legislation concerning human research.

After DNA isolation, the most prevalent variants in the White population of the aforementioned pancreatic susceptibility genes were investigated.^5,6^ Targeted next-generation sequencing of regions including these variants of all ERCP patients was performed in a single run on the MiniSeq system (Illumina, San Diego, CA) and obtained data were analyzed afterward with Sequence Pilot (JSI, Medical Systems, Ettenheim, Germany). Selection criteria involved a >25% variant variation for identifying heterozygous variants and a >75% variant variation for identifying homozygous variants.

The primary outcome was the presence of known genetic variants in *PRSS1* (p.R122H, p.N29I), *SPINK1* (p.N34S, p.P55S), and *CFTR* (p.F508del, p.R117H, p.R75Q) between patients that developed PEP versus patients who did not develop PEP.

We screened 1022 patients of whom 603 received a buccal smear kit. The response rate was 84.4% (509 patients). Unfortunately, 75 refused to participate, and 86 patients were excluded during data collection. Ultimately, 348 patients were included in the study: 103 patients in the PEP group and 245 in the non-PEP group (Supplemental Fig. 1, Supplemental Digital Content 1, http://links.lww.com/MPA/B338).

Baseline characteristics did not differ between both groups, except for age and body mass index (BMI). PEP patients had a median age of 61 years and BMI of 28 kg/m^2^, compared with a median age of 64 years (*P*=0.021) and BMI of 27 kg/m^2^ (*P*=0.015) in the non-PEP group (Supplemental Table 1, Supplemental Digital Content 1, http://links.lww.com/MPA/B338).

The most common indication for ERCP was (suspicion of) common bile duct stones [n=263 (76%)]. Patients in the PEP group had longer procedure times (47 vs. 33 min; *P*=0.001), more precut sphincterotomies (28% vs. 8%; *P*<0.001) as well as increased rates of pancreatic duct contrast injections (37% vs. 16%; *P*<0.001) and pancreatic duct cannulations (66% vs. 29%; *P*<0.001). In addition, NSAIDs were administered more frequently after ERCP in patients who developed PEP (28% in the PEP group vs. 5% in the non-PEP group; *P*<0.001).

DNA amplification rate was 76% with 68% (236/348) of patients having DNA of sufficient quality to analyze all aforementioned genetic variants. In this study, no associations were found between variants in *PRSS1* (p.R122H, p.N29I), *SPINK1* (p.N34S, p.P55S), and *CFTR* (p.F508del, p.R117H, p.R75Q) and the development of PEP (Table 1). Notably, none of the patients were carriers of variant R122H in *PRSS1*. Furthermore, an analysis of the high-risk population, defined as precut sphincterotomy, unintentional PD cannulation, unintentional injection of contrast into the PD, or more than 10 CBD cannulation attempts, also revealed no significant associations. The incidence of genetic variants is similar to the normal population, except for *CFTR* R117H. We found a genetic variant of *CFTR* R117H in 3.6% of the non-PEP patients versus 0.2% described in literature,^8^ but no significant difference was found between the PEP and non-PEP group.

**TABLE 1:** Genetic Variants in PEP Versus Non-PEP Group

	PEP-group	Non-PEP group	*P*
N34S *SPINK1*	1/71 (1.4)	2/188 (1.1)	1.00
P55S *SPINK 1*	1/71 (1.4)	2/189 (1.1)	1.00
R122H *PRSS1*	0/71 (0.0)	0/184 (0.0)	0.00
N29I *PRSS1*	0/72 (0.0)	1/184 (0.5)	1.00
R117H *CFTR*	2/77 (2.6)	7/193 (3.6)	0.74
F508del *CFTR*	1/77 (1.3)	3/193 (1.6)	1.00
R75Q *CFTR*	1/77 (1.3)	2/193 (1.0)	1.00

Data are n (%).

CFTR indicates cystic fibrosis transmembrane regulator; PEP, post-endoscopic retrograde cholangiopancreatography pancreatitis; PRSS1, protease serine 1; SPINK1, serine protease inhibitor Kazal-1.

A previous cohort study with only 30 PEP patients showed no presence of the *SPINK1* N34S mutation.^9^ This study was limited by the small sample size and the limited number of participating hospitals. In contrast, one of the strengths of our study is its multicenter design with over 300 included patients. In addition, we achieved a high participation rate of 84.4%, reducing the risk of selection bias. Furthermore, data collection was performed by 2 independent investigators. However, baseline and ERCP characteristics were collected retrospectively, which is a limitation. Other limitations include a predominantly White study population and lower DNA quality from buccal smears compared with blood samples. Nevertheless, we reached a DNA amplification rate of 75.5%.

In conclusion, our findings indicate that the presence of variants of *PRSS1* (p.R122H, p.N29I), *SPINK1* (p.N34S, p.P55S), or *CFTR* (p.F508del, p.R117H, p.R75Q) are not associated with a higher susceptibility to PEP.

## Supplementary Material

SUPPLEMENTARY MATERIAL

## References

[1] Akshintala VS Kanthasamy K Bhullar FA . Incidence, severity, and mortality of post-ERCP pancreatitis: an updated systematic review and meta-analysis of 145 randomized controlled trials. Gastrointest Endosc. 2023;98:1–6. e12. 37004815 10.1016/j.gie.2023.03.023

[2] Dumonceau J-M Kapral C Aabakken L . ERCP-related adverse events: European Society of Gastrointestinal Endoscopy (ESGE) guideline. Endoscopy. 2020;52:127–149. 31863440 10.1055/a-1075-4080

[3] Baldwin C Zerofsky M Sathe M . Acute recurrent and chronic pancreatitis as initial manifestations of cystic fibrosis and cystic fibrosis transmembrane conductance regulator–related disorders. Pancreas. 2019;48:888–893. 31268981 10.1097/MPA.0000000000001350 PMC6768061

[4] Drenth J Te Morsche R Jansen J . Mutations in serine protease inhibitor Kazal type 1 are strongly associated with chronic pancreatitis. Gut. 2002;50:687–692. 11950817 10.1136/gut.50.5.687 PMC1773221

[5] Hasan A Moscoso DI Kastrinos F . The role of genetics in pancreatitis. Gastrointest Endosc Clin. 2018;28:587–603. 10.1016/j.giec.2018.06.001 30241646

[6] Hegyi P Wilschanski M Muallem S . CFTR: a new horizon in the pathomechanism and treatment of pancreatitis. Rev Physiol Biochem Pharmacol. 2016;170:37–66. 26856995 10.1007/112_2015_5002 PMC5232416

[7] Németh BC Sahin-Tóth M . Human cationic trypsinogen (PRSS1) variants and chronic pancreatitis. Am J Physiol Gastrointest Liver Physiol. 2014;306:G466–G473. 24458023 10.1152/ajpgi.00419.2013 PMC3949028

[8] Karczewski KJ Francioli LC Tiao G . The mutational constraint spectrum quantified from variation in 141,456 humans. Nature. 2020;581:434–443. 32461654 10.1038/s41586-020-2308-7 PMC7334197

[9] Mystakidis K Kouklakis G Papoutsi A . Is post-ERCP pancreatitis a genetically predisposed complication? Gastroenterol Res Pract. 2012;2012:473960. 22934106 10.1155/2012/473960 PMC3426223

