# Supplementary material for: Genetic Variants in SPINK1, PRSS1, or CFTR Are Not Associated With The Development of Post-ERCP Pancreatitis
Source: Pancreas. 2025 Jan 16;54(5):e482–3. doi: 10.1097/MPA.0000000000002465 (PMC12052084; doi:10.1097/MPA.0000000000002465)
Supplement: SUPPLEMENTARY MATERIAL [file mpa-54-e482-s001.docx]

**Supplementary Figure 1 |** Flowchart of inclusion


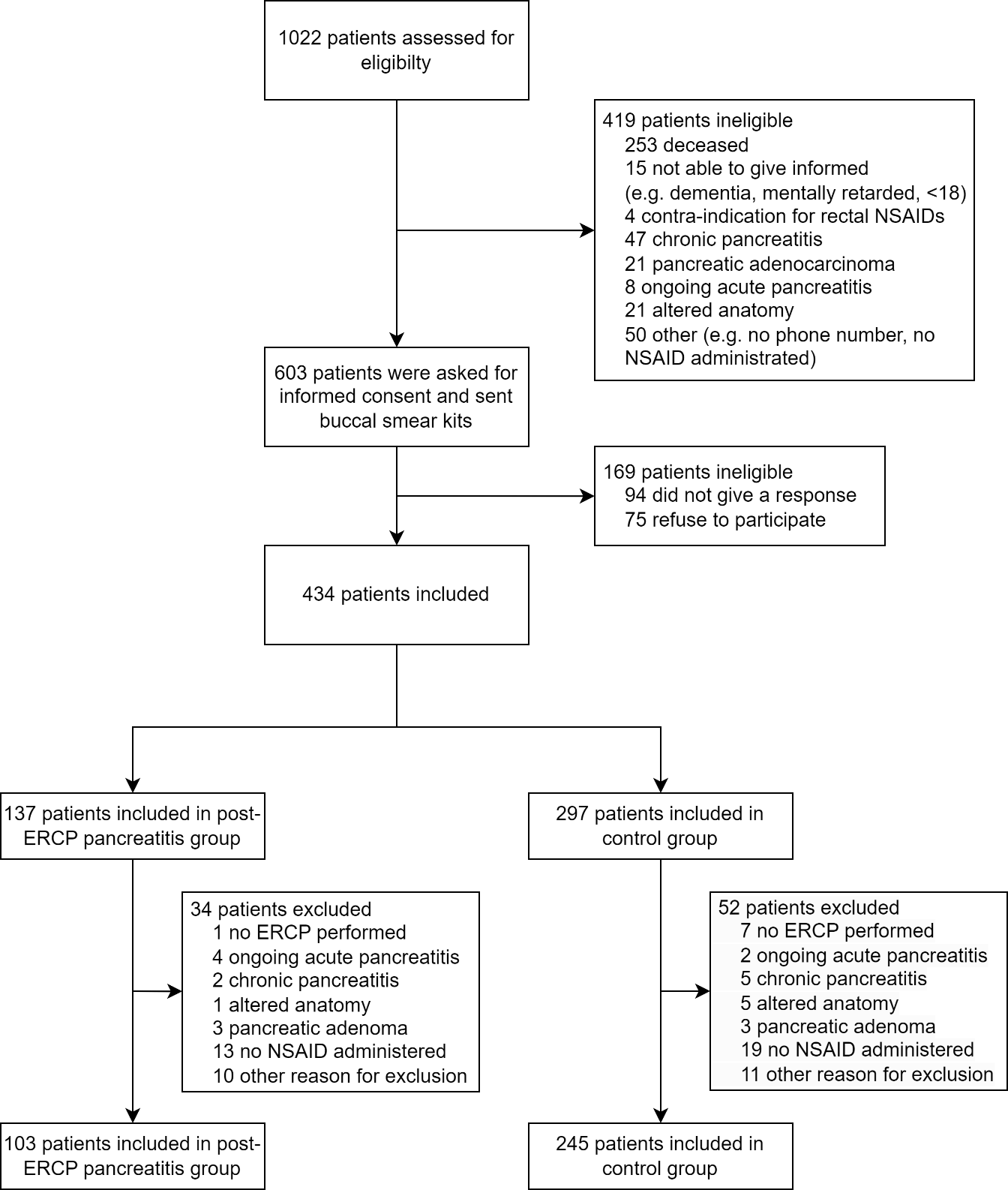


**Supplementary Table 1 |** Baseline characteristics of patients who did develop post-ERCP pancreatitis versus who did not develop post-ERCP pancreatitis.

|  | PEP group (n=103) | Non-PEP group (n=245) | P-value |
| --- | --- | --- | --- |
| Age (years) | 61 (45-70) | 64 (54-72) | 0.021 |
| Sex (female) | 64 (62%) | 137 (56%) | 0.284 |
| Caucasian race | 101 (98%) | 241 (98%) | 1.000 |
| Body-mass index (kg/m^2^) ^(n=100/231)^ | 28 (25-32) | 27 (24-29) | 0.015 |
| ASA class on admission ^(n=103/244)^ |  |  | 0.768 |
| I: healthy status | 28 (27%) | 59 (24%) |  |
| II: mild systemic disease | 59 (57%) | 138 (57%) |  |
| III: severe systemic   disease | 16 (16%) | 46 (19%) |  |
| IV: severe systemic disease that is  constant threat to life | 0 | 1 (0.4%) |  |
| History of acute pancreatitis | 7 (7%) | 22 (9%) | 0.501 |
| ERCP indication |  |  | 0.240 |
| Common bile duct stones without cholangitis | 67 (65%) | 142 (58%) |  |
| Common bile duct stones with cholangitis | 9 (9%) | 45 (18%) |  |
| Metastatic cancer | 0 | 3 (1%) |  |
| Cholangiocarcinoma | 3 (3%) | 4 (2%) |  |
| Postoperative bile leak | 5 (5%) | 14 (6%) |  |
| Sphincter of Oddi dysfunction | 5 (5%) | 5 (2%) |  |
| Bile duct stenosis (unspecified) | 5 (5%) | 11 (5%) |  |
| Other | 9 (9%) | 21 (9%) |  |
| Timing NSAIDs |  |  | <0.001 |
| Before ERCP | 74 (72%) | 232 (95%) |  |
| After ERCP | 29 (28%) | 13 (5%) |  |
| ERCP duration (minutes) ^(n=46/131)^ | 47 (30-66) | 33 (20-50) | 0.001 |
| Conclusion of ERCP |  |  | 0.101 |
| Goal achieved | 68 (66%) | 180 (74%) |  |
| No abnormalities | 12 (12%) | 31 (13%) |  |
| Papilla not reached | 0 (0%) | 1 (0.4%) |  |
| Cannulation CBD failed | 15 (15%) | 14 (6%) |  |
| Cannulation CBD achieved, goal failed | 6 (6%) | 16 (7%) |  |
| Other | 2 (2%) | 2 (1%) |  |
| Biliary sphincterotomy ^(n=102/245)^ | 66 (65%) | 179 (71%) | 0.120 |
| Precut sphincterotomy ^(n=102/245)^ | 28 (28%) | 20 (8%) | <0.001 |
| Pancreatic duct contrast injection (unintentional) ^(n=96/244)^ | 35 (37%) | 40 (16%) | <0.001 |
| Pancreatic duct cannulation (unintentional) ^(n=98/245)^ | 65 (66%) | 70 (29%) | <0.001 |
| Successful pancreatic duct stent placement (after unintentional pancreatic duct cannulation) ^(n=65/70)^ | 22 (34%) | 16 (23%) | 0.156 |
| Pancreatic sphincterotomy | 6 (6%) | 7 (3%) | 0.217 |

Data are median (IQR) or n (%). n = number of patients in PEP group / number of patients in control group. ASA = American society of Anesthesiologists. PEP = post endoscopic retrograde cholangiopancreatography pancreatitis. NSAID = nonsteroidal anti-inflammatory drugs. CBD = common bile duct
